# Ureteral access sheath. Does it improve the results of flexible ureteroscopy? A narrative review

**DOI:** 10.1590/S1677-5538.IBJU.2024.9907

**Published:** 2024-03-10

**Authors:** José Agustin Cabrera Santa Cruz, Alexandre Danilovic, Fabio Carvalho Vicentini, Artur Henrique Brito, Carlos Alfredo Batagello, Giovanni Scalla Marchini, Fabio César Miranda Torricelli, William Carlos Nahas, Eduardo Mazzucchi

**Affiliations:** 1 Universidade de São Paulo – USP Hospital das Clínicas da Faculdade de Medicina São Paulo SP Brasil Divisão de Urologia - Hospital das Clínicas da Faculdade de Medicina da Universidade de São Paulo – USP, São Paulo, SP, Brasil

## INTRODUCTION

The use of a ureteral access sheath (UAS) in flexible ureteroscopy (FURS) has been a topic of debate and extensive research over the years. This medical device, introduced in 1974 by Hisao Takayasu and Yoshio Aso, was designed to facilitate direct kidney access during Retrograde Intrarenal Surgery (RIRS), a procedure commonly used for treating kidney stones. UAS comes in various sizes and designs, offering features like hydrophilic coatings, anti-kinking properties, multiple working channels, and suction mechanisms. The choice of UAS depends on the specific clinical scenario, including stone size, ureteral access challenges, and operative conditions. Several studies have explored the impact of UAS on flexible ureteroscopy outcomes. While some findings suggest advantages, such as improved visibility, multiple entries, and reduced intrapelvic pressure, controversies persist. The type of UAS used, its caliber, and the presence of innovative features like suctioning mechanisms can impact surgical success rates, operative duration, and postoperative complications. One significant advantage associated with UAS usage is a potential reduction in infectious complications, including fever, urinary tract infections, and sepsis. However, concerns about ureteral trauma and complications associated with UAS persist, and the decision to use UAS should be carefully considered on a case-by-case basis. Recent advancements in laser technologies, the miniaturization of digital ureteroscopes, and the introduction of devices equipped with pressure-measuring and aspiration technology may reshape the landscape of flexible ureteroscopy, potentially enabling its application in surgeries involving larger volume calculi. Therefore, the use of UAS may play a significant role in certain cases. The aim of this article is to review the current literature on the use of UAS, advantages, risks, and future perspectives.

## MATERIALS AND METHODS

A search was conducted on PubMed and Embase from June to July 2023. The search terms used to obtain the required information were as follows: "Ureteral access sheath," "ureteric access sheath," and "flexible ureteroscopy,"

Articles published from 2013 to 2023 were included. A total of 472 articles were identified, with 421 articles being excluded due to their lack of relevance, pertaining to the pediatric population, or not being available in English, Portuguese, or Spanish.

**Figure 1:**
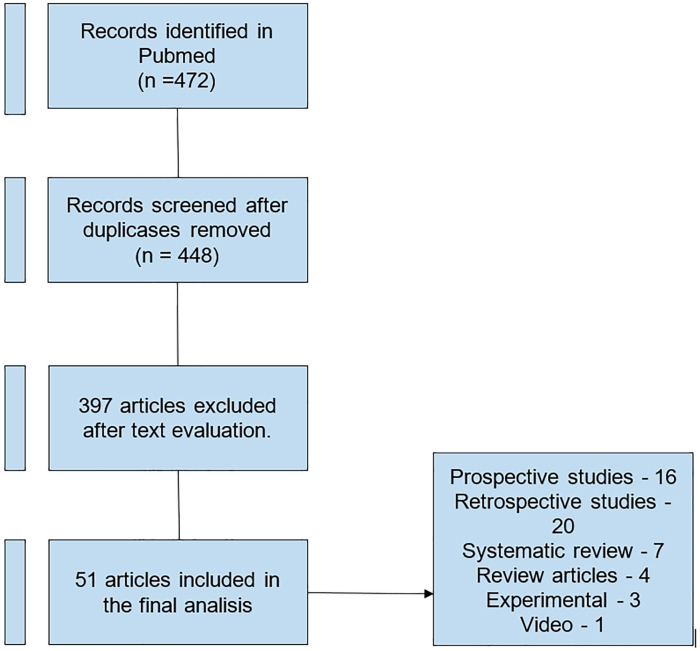
Flowchart used in literature review.

Finally, 50 articles were included in this review, as shown in our flowchart (Figure-1).

### The ureteral access sheath

The ureteral access sheath, developed by Hisao Takayasu and Yoshio Aso in 1974, is a medical device frequently employed during flexible ureteroscopy. Its primary purpose is to enhance direct kidney access during RIRS. The utilization of the UAS has sparked debates owing to its potential benefits and drawbacks. It has demonstrated advantages such as improved visibility, multiple entry points, effective removal of fragmented stones, and the reduction of intrapelvic pressure (1, 2).

There are currently no clear indications in the literature for the use of UAS; however, as we will discuss in this review, the utilization of UAS can be advantageous in specific situations.

### Types of UAS

**Figure 2:**
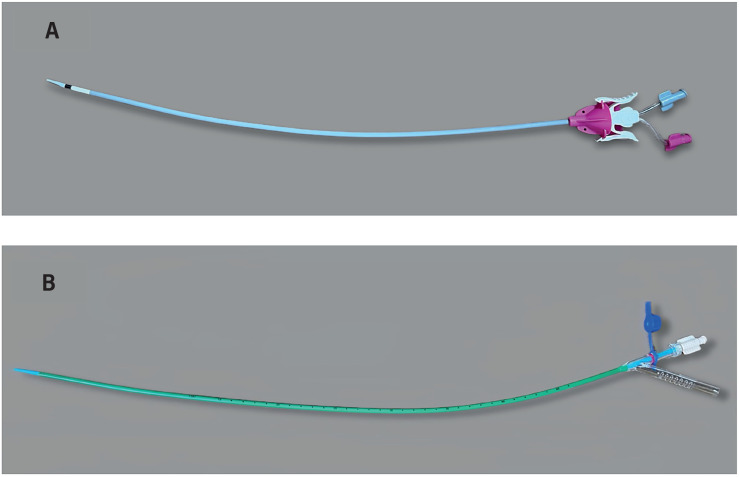
A) Bi- Flex Evo™ UAS. B) ClearPetra® Flexible tip-bendable suction UAS.

UAS come in various sizes and designs. Some are hydrophilic-coated to facilitate insertion, while others have additional features such as anti-kinking properties, multiple working channels, or suction mechanisms (Figure-2) (3). The most used calibers are 10-12Fr (where 10 Fr is the internal diameter and 12 Fr is the external diameter), 11-13Fr, 12-14 Fr, or 14/16 Fr. (Table-1). Some studies have conducted comparisons between UAS of the same and different calibers (4). In a prospective randomized trial conducted by Elsaqa et al., two types of UAS of 12-14 Fr (Boston Scientific Navigator and Cook Flexor) were compared. The study concluded that both UAS demonstrate similar levels of safety and effectiveness when used in flexible ureteroscopy and RIRS (3). Huettenbrink et al. conducted a prospective study to compare two groups in which UAS of 10/12 Fr and 12/14 Fr were used. The study demonstrated that there were no differences in median surgery duration, overall complication rate, and hospitalization. There were no significant differences in stone-free rates (97.9% vs. 92.7%, p=0.37). The duration of laser lithotripsy using Holmium laser was higher in the group of UAS with smaller caliber (1.9 min vs. 3.8 min p<0.01), without showing an increased risk for clinical complications (4). Regarding the use of UAS with different calibers in unstented ureters, in a retrospective cohort study, the use of smaller (9.5/11.5Fr) vs. larger-caliber (12/14Fr) UAS was compared. No significant differences were found in terms of SFR and the rate of complications (4). The insertion success rate can vary depending on the caliber of UAS, as demonstrated by Li et al. (5) They found that the utilization of 10/12 Fr UAS resulted in a higher insertion success rate (91.2% vs. 86.9%, P = 0.006) and lower severity of ureteral wall injury (80.1% vs. 85.2%, P < 0.001) compared to 12/14 Fr UAS. The SFR was similar in both groups. Furthermore, while there was no significant overall difference in SIRS incidence, the 10/12 Fr group saw a significant increase in cases of SIRS when dealing with stones larger than 2 cm. In another similar study, outcomes of 12/14 Fr and 14/16 Fr UAS were compared. There were no significant differences in terms of ureteral injury rates, complications, or SFR. Furthermore, it was observed that the use of a 14/16 Fr UAS improved operative efficiency (2.11 vs. 1.62 mm2/min; p = 0.01) (6).

**Table 1:** Main ureteral access sheaths used.

Company	Model	Internal/external diameter (Fr)	Length (cm)
Cook	Flexor	9,5/11,5- 10,7/12,7- 12/14 -14/16 Fr	20, 28, 35, 45, 55 cm
Allwin	U- flex	9.5/11,5- 10/12-10.7/12,7- 12/14 Fr	35, 38, 45 cm
Boston Scientific	Navigator	11/13 - 12/14 - 13/15 Fr	28, 36, 46 cm,
Urolline	Urolline	11/13 - 12/14 Fr	35, 45 cm
Coloplast	Retrace	10/12 - 12/14 Fr	35, 45 cm
Rocamed	Bi- Flex Evo	10/12 - 12/14	35,45 cm
Well Lead Medical	ClearPetra	10/12 - 11/13 - 12/14 - 13/15 - 14/16	26, 36, 40, 46, 55 cm
Princeton Medical Scientific	Turan	10/12 - 12/14 - 14/16	26,36,41,46,56 cm
BD	Proxis	10/12Fr	25, 35, 45 cm

In conclusion, according to the findings reviewed, the size of UAS did not significantly influence the SFR. Using larger caliber access sheaths may reduce intrarenal pressure and provide improved drainage and efficiency, which can also decrease the risk of postoperative infection or SIRS; however, they may also carry a higher risk of associated ureteral injury and may result in a lower insertion success rate (7).

### Experimental studies

Some studies have been conducted to try to understand the inflammatory response of the ureter and kidney following the use of UAS.

Lildal et al. conducted an experimental study with 22 pigs, examining the response of inflammatory markers in the ureter after the placement of a UAS. This study suggests that the use of a UAS can lead to a significant upregulation of pro-inflammatory mediators in the ureteral wall. This finding may have implications for postoperative pain, drainage, and potential complications related to UAS usage (8).

There are specific markers of renal tubular injury, such as KIM-1, which is shed into the urine after acute kidney damage. KIM-1 is a specific marker and is secreted earliest in tubular injury. Ecer et al. conducted a prospective randomized study to compare the variation of this marker following RIRS. It was found that postoperative 4th-hour urine KIM-1/Cr levels were higher in patients without UAS than in patients with UAS. This leads to the conclusion that the use of UAS during RIRS may involve a reduction in kidney injury, as assessed by KIM-1 (9).

### Predictors for UAS passage

There is no consensus on when not to use UAS. However, some studies demonstrate that the progression of UAS may fail in up to one-fifth of the patients. According to them, some factors can predict the success or failure of UAS passage (10, 11).

Mogilevkin et al. conducted a prospective study involving 248 patients to assess the successful placement of the 14Fr UAS and explore potential predictive factors. It was observed that the passage of the 14Fr UAS failed in 22% of patients. Three independent factors predicting the effectiveness of the insertion of the 14Fr UAS were identified: patient age (odds ratio 1.5); a history of prior procedures on the same side (OR: 9.7); and the presence of a Double-J stent (OR: 21.73) (10).

In terms of predictive factors for failure, in a retrospective study, Hu et al. identified a significant association between the failure of UAS passage and specific factors. The study revealed that male patients, a history duration of less than 15 days, and a smaller diameter of the ipsilateral iliac artery (10.6 mm) were all recognized as predictors of UAS passage failure (11).

These studies suggest that older patients, those with a history of prior procedures on the same side, and those with a prior Double-J stent had a higher likelihood of success in the insertion of the UAS. In contrast, male patients, those with a history duration of less than 15 days, or those with a smaller diameter of the ipsilateral iliac artery have a lower chance of UAS progression.

### Pre-procedural stenting

The prior use of a double J stent is a predictive factor for the passage of UAS. The placement of a preoperative ureteral stent is believed to induce passive dilation of the ureter. This dilation contributes to an increased success rate in the placement of the UAS (12).

Law et al. conducted a systematic review and meta-analysis, evaluating 3831 patients across 14 studies. They found that patients undergoing pre-stenting exhibited higher success rates in UAS insertion compared to their non-pre-stented counterparts, with a relative risk (RR) of 1.09 (95% CI 1.05-1.13, p < 0.00001).

Moreover, the pre-stented group demonstrated superior SFR compared to the non-pre-stented group for residual fragments (RF) with cutoffs at both < 1 mm (RR 1.10, 95% CI 1.02-1.19, p = 0.02) and < 4 mm (RR 1.10, 95% CI 1.04–1.17, p = 0.002).

The study also revealed that pre-stented patients had a lower risk of ureteral injuries during UAS insertion compared to non-pre-stented patients (RR 0.69, 95% CI 0.50 - 0.96, p = 0.03) (13).

Despite these significant advantages, there were no notable differences in other intra and postoperative complications between the two patient groups.

The implications of these findings carry significant weight in shaping clinical decision-making concerning pre-stenting in patients undergoing RIRS. It is crucial to consider the potential benefits, particularly in terms of its facilitation of UAS insertion and the subsequent improvement in SFR outcomes.

However, this decision-making process must be well evaluated, considering both the associated costs and factors related to the quality of life for patients, particularly those associated with stent-related symptoms.

### Previous use of alpha-blockers

Alpha-blockers are frequently employed to enhance the passage of ureteral stones by reducing intraureteral pressure and improving fluid flow (17). They achieve this by inhibiting basal tone, peristaltic activity, and ureteral contractions (14).

Regarding the use of alpha-blockers and the reduction of ureteral injuries, Kim et al. carried out a single-blind prospective study where the administration of Silodosin (8 mg for 3 days before surgery) proved to be more successful in preventing substantial postoperative ureteral damage when compared to the control group (9.3% vs. 27.3%; p = 0.031). Furthermore, within the Silodosin-treated group, there was a notable reduction in postoperative pain (15).

Another advantage is that the prior use of alpha-blockers reduces UAS insertion force, as demonstrated by Koo et al., who found that a 7-day course of tamsulosin reduces the maximum ureteral access sheath insertion force. Furthermore, they suggest that if the force required to insert the UAS exceeds 600 G (assessed by a homemade UAS Insertion Force Measurement Gauge), using a smaller diameter sheath may be considered as an alternative option. Alternatively, the procedure can be terminated, and RIRS with prior stenting can be planned for a later time (16).

### Prior ureteroscopy before UAS placement

Some studies recommend the routine use of semi-rigid ureteroscopy prior to inserting an access sheath. Semi-rigid ureteroscopy enables a gentle dilation of the ureter and provides an assessment of whether it is feasible to place an access sheath and to determine ureteral compliance. A compliant ureter was defined as a ureter with a diameter of at least 14Fr, which readily allowed the passage of a 9.5Fr semi-rigid scope alongside a 3Fr. safety guidewire (17). This step will help us choose which size of UAS to use. The access sheath is only introduced if the ureter allows it (13); Otherwise, it is advisable to place a double-J catheter and postpone the surgery or perform flexible ureteroscopy using a working wire. However, in patients for whom the decision is made to perform the procedure without UAS after failed insertion attempts, there is a higher likelihood of encountering complications and facing challenges in achieving a stone-free status compared to cases where the procedure was initially planned to be performed without a sheath (18).

### Use of fluoroscopy for UAS passage

During surgeries involving the use of radiation, it is ideal to adhere to the ALARA criteria (As Low as Reasonably Achievable) (19). UAS usage has been correlated with increased radiation exposure. However, as reviewed, no established doses have been found to demonstrate risk during the procedure. Also, in the majority of cases (more than 70%), the passage of UAS is performed under fluoroscopic guidance using a guidewire (20).

Regarding the passage of UAS without fluoroscopy, Aghamir et al. introduced a fluoroscopy-free technique. Initially, they conducted a semi-rigid ureteroscopy up to the renal pelvis. Then, they placed a 36 cm, 11/13 Fr access sheath without the obturator over a 7.5 Fr semi-rigid ureteroscope. Subsequently, ureteroscopy was repeated with the guidance of a guide wire until the point where the sheath could be inserted smoothly as if the ureteroscope itself was acting as the guiding rod. This procedure was carried out under direct endoscopic visualization. In 83% of the cases, they successfully advanced the UAS using this method. Concluding that the placement of a UAS could be safely achieved using a semi-rigid ureteroscope with direct visual control. This approach resulted in shorter operative times and eliminated radiation exposure during RIRS (20).

### Intrapelvic pressure

The Intrarenal Pressure (IRP) is the pressure measured in the renal pelvis, and it can be expressed in either centimeters of water (cmH2O) or millimeters of mercury (mmHg). The typical baseline IRP range is 0–20 cmH2O. Conditions such as pyelotubular reflux, pyelovenous backflow, and fornix rupture are associated with different pressure levels ranging from 27 to 95 cmH2O (21).

Patel et al. conducted a prospective study to measure intracalyceal pressures during URS. Pressure measurements were taken in various regions, including the renal pelvis, upper pole, interpolar, and lower pole calyces, with and without the use of a UAS. Intracalyceal pressure showed a significant reduction in each region when a UAS was employed. In comparison to patients with a 12/14Fr UAS, those with a 14/16Fr UAS experienced notably lower pressure in the interpolar calyces (25.3±13.1 vs. 44.0±27.5 mmHg, p=0.03) and lower pole calyces (16.2±3.5 vs. 49.2±40.3 mmHg, p=0.004) (22).

In in vitro studies, it was shown that in the absence of UAS, IRP increased with rising irrigation pressures and exceeded 40 cmH2O, reaching up to 153 cmH2O. However, when a UAS was employed, IRP remained below 40 cmH2O for all irrigation pressures (23).

There is enough supporting evidence to validate the hypothesis that the utilization of a UAS enhances the outflow of irrigation during flexible ureteroscopy. Although the level of evidence remains low, it suggests that UASs have the potential to reduce intrapelvic pressure to levels below 30 cm H2O (or 22 mmHg), which is essential for maintaining a safe pressure range during interventions involving forced irrigation (27).

### Intrarenal temperature

Higher laser power levels are associated with elevated temperatures that could potentially harm the kidney. According to the reviewed literature, there is no consistent evidence that the UAS reduces intrarenal temperature. However, it is known that increasing either the inflow or outflow of irrigation can help mitigate these potential risks. Therefore, the use of a UAS could be beneficial (26, 27).

### Outcomes (stone free rate)

The SFR is one of the primary objectives of surgery for stones; however, the results regarding SFR associated with the use of UAS are quite heterogeneous (28). In a series of studies evaluating the outcomes of flexible ureteroscopy with and without the use of UAS, various aspects were explored:

Traxer et al. conducted a prospective multicentric study involving 2,239 patients. The SFR was higher in patients who used UAS (75% vs. 50%), but this difference was not clinically significant (P = 0.604). However, it's important to note that the SFR was measured using non-contrast CT scans in only a subset of patients (29).

Meier et al. conducted a retrospective study involving 5,316 URS procedures. They found that UAS usage was noted in 37.7% of patients with significantly larger stone sizes. The study revealed that the utilization of UAS during ureteroscopy did not lead to a higher SFR. Instead, it was correlated with an elevated occurrence of unscheduled visits to the emergency department and hospitalizations within the 30 days following the surgical procedure (30).

Lima et al. conducted a prospective study involving 338 patients who underwent FURS for kidney stones. The use of UAS was associated with a longer operative duration and a SFR of 88% compared to 94% without UAS, with no statistically significant difference. Notably, the patients who used UAS were those with multiple or larger stones (31).

In a prospective randomized controlled trial conducted by Singh et al., the effect of UAS on the outcome of RIRS was compared. No significant differences were found in terms of SFR and the degree of postoperative pain. Complications were more common in the UAS group but did not reach statistical significance (32).

Sari et al. conducted a retrospective comparative analysis involving 1,808 RIRS procedures, utilizing UAS in 1,489 of them. The success rate exhibited significant differences, with 88.2% for UAS and 81.2% for other techniques. The operation time was longer when UAS was utilized (42.9 minutes vs. 46.9 minutes). Similar findings were discovered in a prospective study by Traxer et al., where the operation time was longer in the UAS group, possibly due to the larger stone size in this group in both studies. Additionally, Ozimek et al. found that UAS usage was associated with extended surgical procedure duration, a higher likelihood of hospital stays exceeding 48 hours, a more frequent occurrence of postoperative SIRS, and reduced rates of postoperative SFR (60.20% vs. 78.92%) (33, 34).

### Complications of UAS use Risk of infections

Recent studies have investigated the impact of UAS usage on infectious complications during URS procedures across multiple centers.

Traxer et al. conducted a prospective study involving URS patients from various centers. The research gathered data from consecutive patients treated globally over a 1-year period. 1494 patients underwent treatment with a UAS, while 745 received treatment without a UAS. They found that employing a UAS resulted in a significant reduction in infectious complications. In the UAS group, the rates of fever, urinary tract infections, and sepsis were 28.6%, 18.6%, and 4.3%, respectively. In contrast, the non-UAS group experienced higher rates of these complications, with rates of 39.1%, 23.9%, and 15.2%, respectively (29).

In another retrospective study by Villa et al., which analyzed data from 451 ureteroscopy procedures in 369 patients, 11.5% experienced fever, 2.2% had sepsis, and 1.3% developed septic shock. Interestingly, 70% of urosepsis cases and 83.3% of septic shock events occurred in patients who were not treated with UAS (35).

Furthermore, a prospective randomized trial conducted by Bozzini et al. involving 181 patients in two groups (with and without UAS) demonstrated that the UAS group had a lower rate of postoperative fever, urosepsis, and positive cultures. The overall postoperative infection rate was significantly lower in the UAS group at 16.3%, compared to 37.1% in the non-UAS group (p = 0.03) (36).

In summary, the use of UAS is associated with a reduced incidence of infectious complications, including fever, UTIs, and sepsis, in patients undergoing URS procedures.

### Postoperative pain

The relationship between the use of UAS and postoperative pain is complex and may vary across different studies. While some studies suggest an association between UAS usage and increased postoperative pain and complications, others did not find significant differences.

In a retrospective study when outcomes were compared between the use and non-use of UAS, a significant association was observed between emergency department visits and the use of UAS, with postoperative pain being the most frequent cause (48%) (37).

In a prospective study, postoperative pain following RIRS was evaluated. All patients used a UAS. It was found that the size of the ureteral access sheath was not statistically associated with postoperative pain (p>0.05). On the other hand, the intraureteral dwell time of the ureteral access sheath during the operation (sheath time) was significantly higher in the group that experienced greater postoperative pain, with 46.57 minutes compared to 41.54 minutes (38).

Damar et al. conducted a prospective study involving 60 patients, finding that postoperative pain, as measured using the Visual Analog Scale (VAS), was slightly higher in patients who did not use a UAS compared to those who did (5.33 vs. 4.13, p = 0.064) (39).

### Ureteral trauma/stricture

Ureteral trauma is one of the potential complications associated with the use of UAS. In the study by Bozzini et al., ureteral lesions were found in 41.3% of the cases, with the majority being of grade 1 (36). Similarly, Traxer et al. conducted a prospective study with 359 patients to evaluate ureteral injuries following the placement of UAS. In this study, ureteral wall lesions associated with UAS were detected in 46.5% of patients, with most falling into the low-grade injuries category (grade 1), comprising 86.6% of cases. Grades 2, 3, and 4 injuries were observed in 10.1%, 3.3%, and 0.0% of patients, respectively.

Interestingly, the frequency of severe injuries was comparatively reduced among females compared to males (p < 0.024) and in younger patients versus older patients (p < 0.018). No relationship was found between body mass index, a medical history of diabetes mellitus, cardiovascular diseases, or abdominopelvic radiation (40).

Regarding the formation of ureteral strictures following the use of UAS, a prospective study demonstrated that high-grade ureteral lesions after the placement of a ureteral access sheath do not result in clinically significant outcomes during intermediate-term follow-up. The stricture rate is similar to that observed in cases without visible injuries, standing at 1.8% (41).

In another study conducted by Shvero et al., a retrospective evaluation was performed on 165 patients who underwent the use of both small and large caliber UAS in an unstented ureter. The study did not find any ureteral stricture following the use of UAS. However, it's important to note that the average follow-up time in the study was only 115 days (42).

These studies demonstrate that, despite a considerable percentage of ureteral trauma following UAS placement, the vast majority of these injuries are mild (43). Additionally, no association was found between ureteral stricture and the use of the UAS. However, few studies are providing long-term follow-up to assess such late complications (44).

### UAS with suction

**Figure 3:**
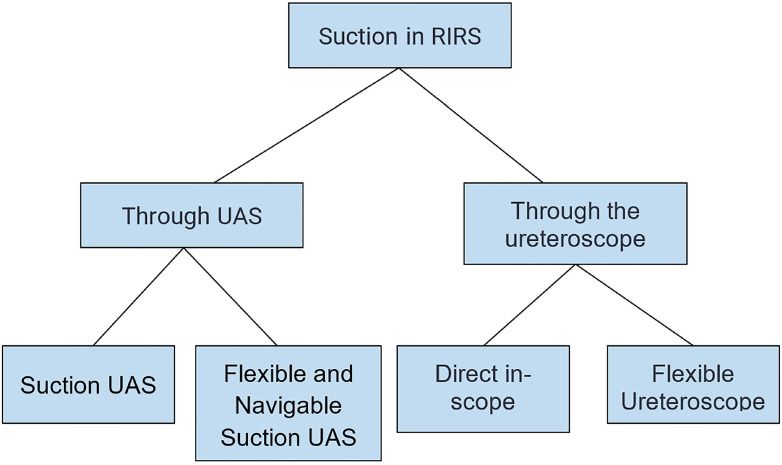
Main suction techniques used in RIRS. Adapted from Giulioni et al. (50).

In recent years, suction mechanisms have been introduced into the UAS to enhance efficacy during RIRS and reduce complications (45) (Figure-3). Recent studies have compared the use of UAS with and without suction.

Zhu et al. compared the use of a suctioning UAS versus a traditional UAS during RIRS. The suctioning UAS group had a significantly higher success rate in the immediate postoperative period (82.4% vs. 71.5%; P = 0.02). The stone-free rate at one month was higher in the suctioning UAS group, although this result was not statistically significant. The suctioning UAS group was associated with a lower complication incidence compared to the traditional UAS group (24.8% vs. 11.5%; P < 0.001). It was also linked to a shorter operative time (49.7 ± 16.3 minutes vs. 57.0 ± 14.0 minutes; P < 0.001) (46).

In a similar study, Qian et al. Evaluated retrospectively 444 patients to assess differences between UAS with and without suction. The SFR on the first day in the suctioning group was higher than that in the non-suctioning group (86.4% vs. 71.6%; P = 0.034). However, no significant difference was observed in the success rates one month postoperatively between the two groups (82.7% vs. 88.9%; P = 0.368). The suctioning group had a significantly lower incidence of postoperative fever (3.70%) compared to the non-suctioning group (14.8%) p= 0.030. Furthermore, the occurrence of postoperative SIRS was lower in the suctioning UAS group (1.23% vs 12.3% p=0.012) (47).

More recently, Gauhar et al. showed the use of the Flexible and Navigable Ureteric Access Sheath (FANS). This device allows for the mobilization of the UAS through various calyces due to its flexibility. The retrospective study included 45 patients, revealing that 71.1% had a 100% SFR immediately post-operation, which improved to 93.3% at the 3-month follow-up (48).

Another retrospective study conducted by Liang et al. presented an initial series of 224 cases where RIRS was performed using FANS. The primary outcomes included an immediate SFR of 76.8% and 97.3% at 30 days postoperatively. The majority of cases (95.5%) achieved success in a single session, with an average operative time of 69.2 ± 65.2 minutes. The postoperative complications were minimal, with only two patients experiencing fever and no unplanned readmissions.

It is interesting to mention that, in patients who had lower calyx stones, the immediate SFR was considerably lower than in patients without lower calyx stones (68.6% vs. 88.9%, P < 0.05). In addition, the use of baskets was significantly higher in these patients (50% vs 6.7%) (49).

It is important to consider that in the context of treating lower calyx stones, the infundibulopelvic angle (IPA) plays a crucial role, as demonstrated by Danilovic et al. in a prospective study. In this study, an IPA measuring below 41° was associated with an increased likelihood of residual fragments after undergoing RIRS for kidney stones up to 20 mm (50). Furthermore, the maximal deflection of the tip bendable UAS is limited and depends on the flexibility of the ureteroscope. Therefore, one of the potential drawbacks of the flexible UAS is in the treatment of lower calyx stones, especially when associated with a steep IPA.

The reviewed evidence supports the use of aspiration during flexible ureteroscopy as an effective strategy to enhance the removal of kidney stone fragments and ultimately increase SFR. Among the suction modalities, the use of UAS with suction exhibited lower complication rates, shorter operative times, and reduced incidences of postoperative fever and SIRS. They can control intrarenal pressure, thereby improving visualization and stone fragment removal without the requirement for retrieval baskets (51).

These findings are encouraging for streamlining and enhancing the kidney stone treatment process. However, it is important to consider some limitations, such as sample size, types of studies, and the absence of long-term data.

## CONCLUSIONS

The use of UAS improves the results of flexible ureteroscopy in specific scenarios, such as those involving challenging ureteral access or for patients with kidney stones who have a higher risk of developing infectious complications (patients with immunosuppression, previous urinary infections, or double-J catheters). Additionally, it can be considered as an option when dealing with challenging operative conditions where visibility is compromised due to insufficient irrigation fluid outflow. Furthermore, the adoption of UAS is directly correlated with stone size, making it particularly useful for larger stones or when multiple stones are present.

## PERSPECTIVES

The future outlook for the use of Ureteral Access Sheaths in urology is undeniably promising, marked by a convergence of technological advancements that are poised to redefine stone management strategies. New laser technologies, including high-powered Holmium, Thulium fiber laser, and Thulium YAG, are at the forefront of this evolution, promising enhanced precision and efficacy in stone fragmentation.

The miniaturization of ureteroscopes represents a pivotal shift, enabling greater maneuverability and reducing invasiveness during procedures. The incorporation of pressure-measuring technologies and the introduction of aspiration capabilities, both within the endoscope and the sheath, further enhance procedural control and real-time feedback.

The advent of flexible sheaths adds an extra layer of innovation, facilitating navigation through intricate anatomical structures. This flexibility is particularly crucial in the context of utilizing flexible ureteroscopy for larger stone volumes, where the aid of UAS becomes indispensable.

The ongoing evolution of these technologies is complemented by the entrance of artificial intelligence (AI) into urological procedures. The potential integration of AI in planning, execution, and postoperative assessment may optimize stone targeting, improve navigation, and elevate overall treatment precision.

In essence, the future of UAS in urology appears dynamic and multifaceted, with innovations converging to create a more tailored and efficient approach to stone management. The synergy of high-powered lasers, miniaturized instrumentation, advanced pressure-measuring capabilities, the use of aspiration, improved maneuverability, and the potential integration of AI positions the UAS as a crucial tool in the evolving landscape of urological interventions. This is particularly evident in the context of flexible ureteroscopy for larger stone burdens.
